# A Phage Therapy Guide for Clinicians and Basic Scientists: Background and Highlighting Applications for Developing Countries

**DOI:** 10.3389/fmicb.2020.599906

**Published:** 2021-02-11

**Authors:** Ali Khalid, Ruby C. Y. Lin, Jonathan R. Iredell

**Affiliations:** ^1^Centre for Infectious Diseases and Microbiology, Westmead Institute for Medical Research, Sydney, NSW, Australia; ^2^Faculty of Medicine and Health, School of Medical Sciences, The University of Sydney, Sydney, NSW, Australia; ^3^School of Medical Sciences, University of New South Wales, Sydney, NSW, Australia; ^4^Westmead Hospital, Western Sydney Local Health District, Sydney, NSW, Australia

**Keywords:** bacteriophage, developing countries, antibiotic resistance, mortality, disease burden

## Abstract

Approximately 10% of global health research is devoted to 90% of global disease burden (the so-called “10/90 Gap”) and it often neglects those diseases most prevalent in low-income countries. Antibiotic resistant bacterial infections are known to impact on healthcare, food security, and socio-economic fabric in the developing countries. With a global antibiotic resistance crisis currently reaching a critical level, the unmet needs in the developing countries are even more striking. The failure of traditional antimicrobials has led to renewed interest in century-old bacteriophage (phage) therapy in response to the urgent need to develop alternative therapies to treat infections. Phage therapy may have particular value in developing countries where relevant phages can be sourced and processed locally and efficiently, breaking specifically the economic barrier of access to expensive medicine. Hence this makes phage therapy an attractive and feasible option. In this review, we draw our respective clinical experience as well as phage therapy research and clinical trial, and discuss the ways in which phage therapy might reduce the burden of some of the most important bacterial infections in developing countries.

## Introduction

**FIGURE 1:**
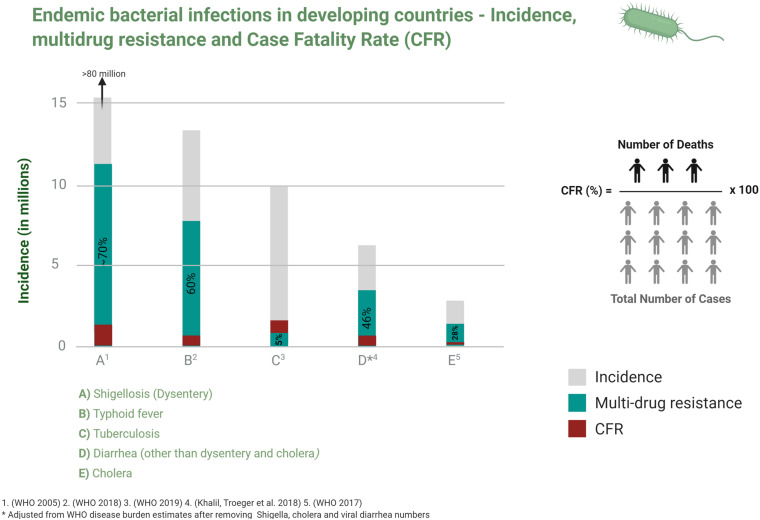
Incidence, multidrug resistance and case fatality ratio of common bacterial infections in developing countries. Data is taken from studies cited in relevant disease sections in the text.

In 1990, the Global Burden of Disease Study (GBD) began to monitor the burden of specific health conditions in populations at national, regional and global levels in order to inform health policies especially in developing countries (Michaud, 2009). Almost two decades on, the 2017 GBD report indicated an improvement in the overall mortality from communicable infections but pointed to the continuing heavy socio-economic and public health burdens in developing countries (GBD Causes of Death Collaborators, 2018; Figure 1).

Infectious diseases disproportionately affect developing countries which, when combined with malnutrition, unhealthy living conditions and unsafe drinking water, drive morbidity and mortality and economic injury. The global expansion of antibiotic resistance (AMR) not only exacerbates this but also threatens to reverse the reductions in mortality and morbidity from endemic infections that are enjoyed in developing countries (Figure 1). A recent report estimated that AMR will contribute an excess of 10 million deaths and a GDP loss of $100 trillion USD by 2,050 if effective measures are not taken to contain it (O’Neill, 2016).

Developing countries have in common limited healthcare systems and fragile economies and are ill-equipped to manage a growing infectious diseases burden despite all efforts from international health and humanitarian organizations (Bhutta et al., 2014).

In 2014, the first World Health Organisation (WHO) global surveillance report on antibiotic resistance showed that > 50% of clinically important bacteria from five of the six WHO regions have resistance against third generation cephalosporins, fluoroquinolones and carbapenems, and attributed 45% of deaths in Africa and South East Asia to multi-drug resistant (MDR) bacterial infections (WHO, 2014). Poor socioeconomic conditions, illiteracy, limited healthcare facilities, and unregulated antimicrobial use in humans and animals are important contributors to undesirable antibiotic resistance trends and their consequences (Aarestrup, 2012; Ayukekbong et al., 2017).

In recognition of this, a consortium of major pharmaceutical companies is creating a $1 billion for-profit venture in support of small biotechnology companies developing mid-stage antibiotics (Silverman, 2020). While a comprehensive and integrated collaboration to antibacterial compounds and vaccines at global level is currently underway (Tong, 2020), the pathway to market access remains a barrier. The golden era of antibiotics continues to fade and there is an urgent need to develop and implement novel therapeutic strategies for infectious (Alanis, 2005).

The century-old science of bacteriophage (phage) therapy was largely neglected after the advent of antibiotics (Summers, 2001) but there remain distinct advantages. Phages are highly specific antibacterial agents that cause much less collateral damage to the microflora than conventional antibiotics that can be applied directly to human tissues without causing harm (d’Herelle, 1931; Weber-Dabrowska et al., 1987; Petrovic Fabijan et al., 2020a) and their abundance means they can be locally sourced, processed and packaged (Nagel et al., 2016).

**FIGURE 2:**
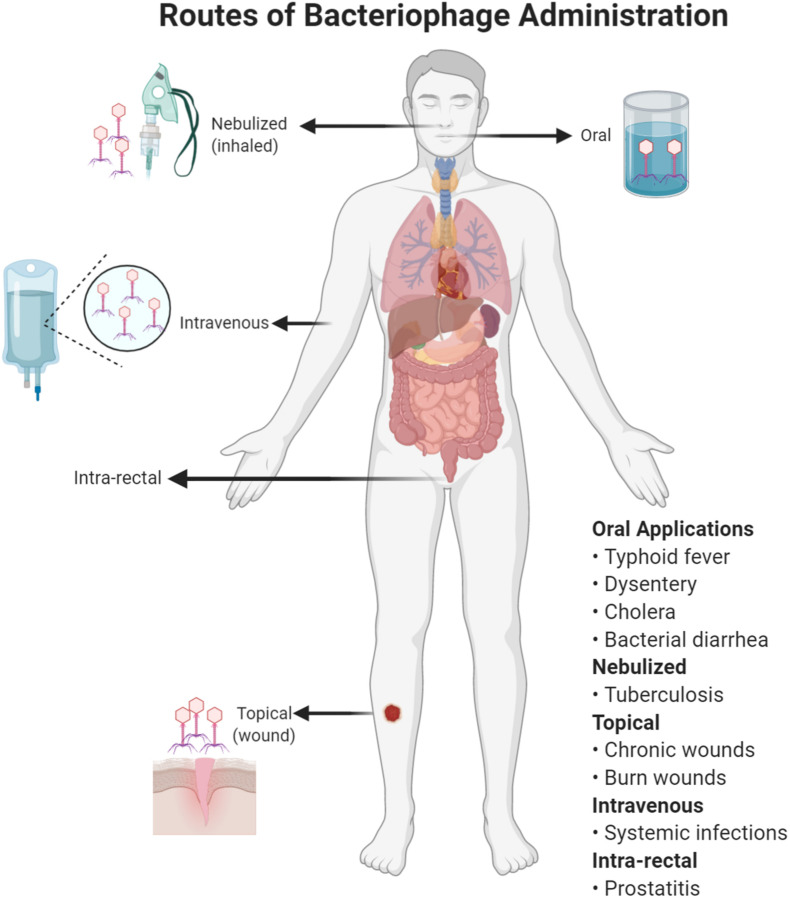
Different routes of bacteriophage administration. The choice of a route will depend on the type of infection treated with phage therapy.

Good Manufacturing Practice (GMP) preparations free of bacterial contaminations (especially lipopolysaccharides) for intravenous (IV) administration are a manufacturing challenge (not just in developing countries) but alternative administration of phages topically and orally (Figure 2) can be effective and feasible (Gill and Hyman, 2010). Here, we discuss some prominent infections for which phage therapy might be considered (Table 1).

**TABLE 1:** Important endemic bacterial infections in developing countries and their possible phage therapy solution.

Disease	*Incidence (in millions)	MDR/XDR	Phage trials		Phage solution	
			Animals	Humans	Prophylaxis	Treatment
Typhoid	11–20	Yes/Yes	Yes	Yes	Yes	Yes
Cholera	2.86	Yes/No	Yes	Yes	Yes	Yes
Shigellosis (dysentery)	>80	Yes/Yes	Yes	Yes	Yes	Yes
Tuberculosis	10	Yes/Yes	Yes	No	Yes	No
Acute bacterial diarrhea other than dysentery and cholera	∼5–5.5	Yes/No	Yes	Yes	Yes	Yes

*The references for these data can be found in relevant sections.

### Typhoid

Typhoid fever is an occasionally fatal systemic infection caused by *Salmonella typhi* and *paratyphi* strains, responsible for 11–20 million cases and 128,000–161,000 deaths globally each year (Ochiai et al., 2008; Mogasale et al., 2014). A new typhoid conjugate vaccine with longer immunity and better safety profile in children <2 years age has recently been approved (Shakya et al., 2019) but currently available vaccines do not provide long-lasting immunity and vaccination has not been widely implemented in endemic countries.

Extensively drug resistant (XDR) *Salmonella typhi* (to first line antibiotics ampicillin, chloramphenicol and trimethoprim-sulfamethoxazole, as well as fluoroquinolones and third generation cephalosporins) is now widespread in countries like Pakistan (Klemm et al., 2018) and is regularly imported to the United States (Chatham-Stephens et al., 2019), Australia (Howard-Jones et al., 2019), Canada (Wong et al., 2019), the United Kingdom (Klemm et al., 2018), and other countries in Europe (Kleine et al., 2017; Engsbro et al., 2019; Lopez-Segura et al., 2019; Procaccianti et al., 2020). An extensively drug resistant *Salmonella typhi* of a different haplotype (H58) from the epidemic strain in Pakistan (H55) has also emerged in Africa (Akinyemi et al., 2015; Phoba et al., 2017) which is responsive only to last-line hospital intravenous carbapenem antibiotics.

Physicians have used phages to treat typhoid for nearly a century (Smith, 1924) and historical anecdotes includes both oral and intravenous therapy (d’Herelle, 1931). Phages were used successfully in major outbreaks in Los Angeles from 1936 to 1949 (Knouf et al., 1946) and Quebec from 1946 to 1949 (Desranleau, 1948, 1949) and phage therapy for typhoid is once more in focus as antibiotics are failing. The widespread incidence of typhoid fever in low-income countries suggests that their natural predator phages should be present in the environment as well but international biobanks are already acting as vital repositories. Phages with lytic activity against an XDR *Salmonella typhi* strain isolated in the Democratic Republic of the Congo (Kakabadze et al., 2018) were identified from the phage library at the Eliava Phage Therapy Center in Georgia, where a Phage Biobank of obligately lytic phages against most common human pathogens is kept. Typhoid fever may be an ideal candidate for “re-introduction” of phage therapy.

### Cholera

**FIGURE 3:**
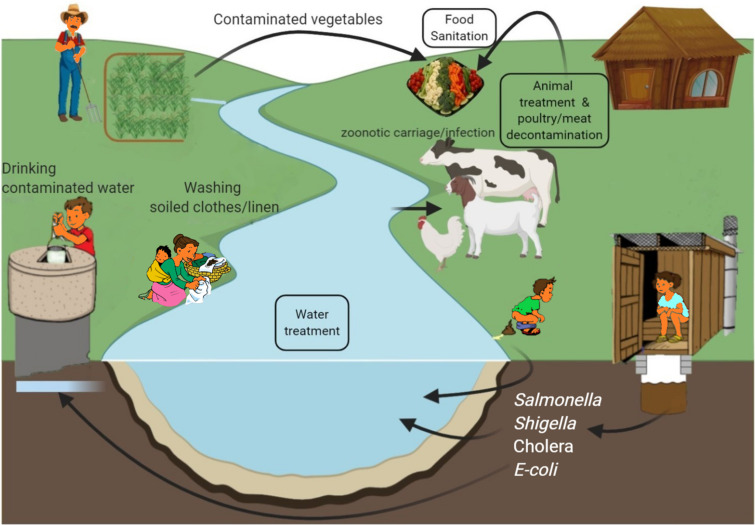
Environmental sources of common bacterial infections in developing countries and possible phage interventions (boxed text) as preventive measure.

Cholera is a self-limiting, rapidly dehydrating secretory diarrheal disease of humans caused by toxigenic strains of the Gram-negative bacterium *Vibrio cholerae*. It is a major cause of mortality and morbidity in developing countries of Asia and Africa and is associated with poor sanitation and lack of clean drinking water (WHO, 2017a; Figure 3), with outbreaks often following war or natural disaster (Gupta et al., 2016). In 2018, nearly half a million cases and 3,000 deaths were reported (WHO, 2018). However, lack of diagnostic facilities, inadequate disease surveillance and fear of adverse effects on trade and tourism may all contribute to significant underreporting and WHO estimated the real case load to be nearly 3 million annually in endemic areas with 95,000 deaths (WHO, 2017a), more than half of these being in children ≤ 5 years old (Ali et al., 2015).

Currently, there are three killed whole-cell oral vaccines prequalified by WHO for use in children > 1 year and adults (WHO, 2017a; Seo et al., 2020) and a global stockpile has been created. Millions of doses have been administered and the WHO Global Task Force on Cholera Control aims to end cholera by 2030 through improved surveillance, vaccination, and implementation of improved water, sanitation, and hygiene in “hotspot” areas to reduce incidence and transmission (Zaman et al., 2020). Despite this, cholera has recently returned to the Americas with ongoing transmission in Haiti (Ganesan et al., 2020; Figure 3).

Management of cholera requires aggressive fluid and electrolyte replacement, but antibiotic treatment may decrease diarrhea by 50% and reduce shedding of viable organisms by days (Harris et al., 2012). Chemoprophylaxis within households may be effective but is not recommended by WHO because of the risk from AMR. Most *V. cholerae* in endemic areas are now resistant to the commonly used antibiotics (Dengo-Baloi et al., 2017; Rijal et al., 2019; Verma et al., 2019; Chatterjee et al., 2020).

An inverse relationship between the presence of virulent cholera phages and *V. cholerae* in environmental water samples coincides with the seasonality of disease occurrence in surrounding populations. This indicates a key role for phages in cholera epidemiology (Faruque et al., 2005) and makes biological control using phages an attractive option. Phages have been used to treat cholera since d’Herelle first linked the decline in mortality during a cholera epidemic in India in the 1920s to phages in stool and began to treat patients with oral vibriophages. He achieved a dramatic reduction in mortality with early use of oral phages (d’Herelle, 1929) and subsequent field trials achieved remarkable results in controlling localized epidemics (Summers, 1993). Several later trials reported successful prophylaxis (Sayamov, 1963) but relatively little effect on duration of established diarrheal illness (Monsur et al., 1970) or pathogen excretion (Marcuk et al., 1971).

Therapeutic effectiveness of cholera phages has also been shown in more controlled experiments using animal models, even with single phages (Bhandare et al., 2019). A “cocktail” of five specific phages given 6 and 12 h before *V. cholerae* challenge in adult rabbits slightly reduced both disease severity and bacterial load (Jaiswal et al., 2013) and another study using three phages in combination up to 24 h before *V. cholerae* challenge prevented infection in infant mouse and rabbit models, without emergence of resistance (Yen et al., 2017). It may be that poor choice of phages and/or trial design have contributed to previous failures and it seems clear that rigorous clinical trials of well-selected vibriophages are warranted.

### Shigellosis (Bacillary Dysentery)

Shigellosis is a major public health problem in low-middle income countries and an important cause of morbidity in industrialized countries (Kotloff et al., 1999). It is generally a self-limiting diarrheal illness of up to 10 days but severe cramps and mucosal bleeding are not uncommon. Complications include sepsis, encephalopathy, hemolytic uremic syndrome and, rarely, intestinal perforation (Khan et al., 2013). Case-fatality rates as high as 28% have been reported in children (Tickell et al., 2017).

Shigella is highly infectious, with very low infectious dose (DuPont et al., 1989) and efficient transmission through fecal-oral route and in contaminated food, water and fomites (Figure 3). Common sources include salads, poultry, milk and dairy products, seafood and vegetables (Ahmed and Shimamoto, 2015) and *Musca domestica*, the common housefly with an affinity for human excrement, has also been incriminated as a mechanical vector (Cohen et al., 1991). In humans, maternal immunity may reduce incidence of *Shigella* infections in the first 6 months of life before immunity matures and becomes protective (Mani et al., 2016). Repeated infections, however, are not unusual because immunity is highly specific and multiple serotypes cause infection. This is most problematic in developing countries during summers and rainfall season (Figure 3) and where over-crowding and unsatisfactory hygienic is common (Puzari et al., 2018).

The four serologically distinguishable *Shigella* species are *S. dysenteriae, S. flexneri, S. boydii*, and *S. sonnei*, all of which cause shigellosis. *Shigella* causes diarrhea among travelers and military personnel from high-income countries (Khalil et al., 2018) and it is increasingly problematic in men who have sex with men communities (Heiman et al., 2014; Baker et al., 2018). Geographical variations and changing *Shigella* epidemiology (Tickell et al., 2017) makes management more difficult (Baker et al., 2018). There are several promising candidate in different stages of development (Walker, 2015) but no licensed vaccines are presently available.

Antimicrobial treatment can shorten the disease course, prevent complications and limit spread of infection through fecal shedding (Williams and Berkley, 2018). As with other endemic infections however, the treatment of shigellosis is complicated by increasing AMR. Currently, WHO recommends fluoroquinolones as first line treatment for all cases of dysentery and third-generation cephalosporins (ceftriaxone) are reserved as a second line or alternative option (WHO, 2005a) because there is high prevalence of resistance against ampicillin, tetracyclines, and sulphonamides. Quinolone, macrolide and third generation cephalosporin resistance is now increasingly widespread (Azmi et al., 2014; Chiou et al., 2016; Chung The et al., 2016) and horizontal transfer of resistance determinants between *Shigella* and related Enterobacteriaceae may be contributing to the alarming increase in frequency of MDR globally (Mandomando et al., 2009; Chang et al., 2011; Gu et al., 2012; CDC, 2013; Aggarwal et al., 2016; Nuesch-Inderbinen et al., 2016; Hussen et al., 2019; Wang et al., 2019; Houpt et al., 2020).

Phages against *Shigella flexneri* may offer an alternative—they have been shown to prevent epithelial cell adhesion and invasion of phage-specific strains as well as other isolates of same species in a human intestinal organoid-derived infection model (Llanos-Chea et al., 2019). Early studies in mouse models showed phages can reach a range of anatomic sites including the brain (Dubos et al., 1943) and that phage therapy delayed up to 4 days can still prevent mortality (Morton and Engley, 1945).

Effective phages are relatively easily isolated, including from environmental water sources during dysentery outbreaks (Doore et al., 2018) and there is a rich history of phage therapy for shigellosis, with large and successful interventions reported since the 1930s (Chanishvili, 2012; Goodridge, 2013). Phages have been used successfully for dysentery prophylaxis (Babalova et al., 1968; Anpilov and Prokudin, 1984) and as biocontrol agents in water (Jun et al., 2016) and food (Zhang et al., 2013; Soffer et al., 2017).

### Acute Bacterial Diarrhea Due to *E. coli*

Enteropathogenic *E. coli* (EPEC), enteroinvasive *E. coli* (EIEC), enteroaggregative *E. coli* (EAEC), enterotoxigenic *E. coli* (ETEC), Shiga toxin-producing *E. coli* (STEC), and diffusely adherent *E. coli* (DAEC) are distinguished on the basis of specific virulence properties (Croxen et al., 2013). EPEC and ETEC are endemic in developing countries where ETEC strains are a major cause of traveler’s diarrhea while STEC causes large outbreaks around the world and, like Shigella, may be complicated by haemorrhagic colitis and haemolytic uremic syndrome (HUS) (Rojas-Lopez et al., 2018). The more than 6 billion cases and 1.5 million deaths in all age groups from diarrheal illness globally (Gregory et al., 2018; Spencer et al., 2018) include both viral and bacterial etiologies, but ETEC is in the top 10, accounting for more than 50,000 deaths in 2016, predominantly in young children in sub-Saharan Africa and South Asia (Khalil et al., 2018).

There is a steady rise in antibiotic resistance among diarrheagenic *E. coli* with many developing countries reporting >70% of isolates to be MDR (Nguyen et al., 2005; GebreSilasie et al., 2018; Zhang et al., 2018). The WHO does not recommend routine use of antimicrobials to treat diarrhea where it is not possible to initially distinguish between etiological agents, because their efficacy is limited (WHO, 2005b) and in order to minimize selection for resistance (Laxminarayan et al., 2013). An oral, live attenuated recombinant vaccine (ACE527) was shown to generate strong immune response against ETEC in human volunteers (Darsley et al., 2012; Harro et al., 2019) but is not yet widely available.

Phage preparations have been used to treat potentially lethal enteropathogenic *E. coli* infection in calves, piglets and lambs (Smith and Huggins, 1982, 1983; Smith et al., 1987) and a large Phase I/II trial for the treatment of pediatric *E. coli* diarrhea established safety of orally administered phages in children but failed to significantly improve symptoms or outcomes (Sarker et al., 2016). Inadequate strain coverage, gastric acid neutralization and low pathogen density (for phage amplification) may be contributors to these unsatisfactory outcomes (Brussow, 2019) and need to be considered in future study designs.

### Foodborne Diseases

Foodborne diseases (FBD) are defined as any illness caused by the ingestion of contaminated food or drink. The FBD disease spectrum ranges from gastrointestinal symptoms (most common) to debilitating chronic conditions including neurological and immunological disorders as well as multi-organ failure, cancer, and death (Grace, 2015). Until recently, data on the incidence of FBD and its associated costs were mostly limited to high-income countries as many developing countries lack reliable data on the incidence of FBD.

The annual global incidence of FBD was recently estimated to exceed 600 million cases and 420,000 deaths annually (WHO, 2015), largely experienced in low-middle income countries (LMICs) with less well developed food safety and regulatory and reporting systems (Grace, 2015). Total productivity loss is estimated at more than US$95 billion in LMICs with another $15 billion USD spent on treatment (World Bank Group, 2018) and biological pathogens are the most important food safety risks in developing countries (Käferstein, 2003). Rapid urbanization is a key factor in developing countries with marked increases in food establishments and outlets numbers, inadequate knowledge in food handlers, lack of clean water, use of untreated human and animal waste in agriculture, suboptimal refrigeration, and poor personal hygiene (Käferstein et al., 1997).

*Salmonella, Campylobacter, Enterohemorrhagic E. coli* (EHEC) and *Listeria* are among the most common bacterial pathogens implicated in FBD (Havelaar et al., 2015). Multiple interventions are used to reduce contamination of foods with biological pathogens (Vikram et al., 2020) but all have limitations. There is an increased need for “natural” antimicrobial alternatives for food decontamination and preservation and many studies have established the usefulness of phages, including decontamination of processing surfaces (Moye et al., 2018). In addition to the appeal as an “organic” choice, phages have the advantages of high specificity, self-replication, co-evolution with their bacterial hosts, low toxicity, relatively inexpensive manufacturing and high tolerance of the conditions common in food processing and storage (Sillankorva et al., 2012).

Phages have long been recognized as a normal part of the food ecosystem (Whitman and Marshall, 1971; Hudson et al., 2005). *Listeria monocytogenes*, often associated with fresh or minimally processed foods such as dairy products and salads, was an early target for food sanitization attempts and their effectiveness in food products is well described (Amy Strydom, 2015). The first commercial phage biocontrol application against *Listeria* contamination for food safety was approved by FDA in 2006 (ListShield^TM^) and a Generally Recognized as Safe (GRAS) designation was issued to another *Listeria-*specific phage biocontrol product (Listex^TM^) later that same year. Since then, many other phage applications have been introduced into the market successfully (Vikram et al., 2020) and other pathogens such as *Campylobacter* may soon follow (Sillankorva et al., 2012). There are approved food safety phage applications against *Salmonella*, *enterohaemorrhagic E- coli* and *Shigella* (Moye et al., 2018), which constitute the majority of foodborne diseases of bacterial origin. Food sanitation by phage biocontrol should remain a high priority research and development agenda.

### Tuberculosis

Tuberculosis (TB) is an airborne infectious disease caused by *Mycobacterium tuberculosis* complex. It is primarily a disease of the lungs but can disseminate to affect other parts of the body. TB is a leading cause of global mortality and the highest among all infectious diseases, including HIV/AIDS. WHO reported an estimated 10 million new cases of TB with 1.2 million deaths in HIV-negative people and more than 250,000 deaths among HIV-positive people in 2018 (WHO, 2019). Developing countries account for more than two thirds of the global incidence of TB, with most high-income countries reporting less than 10 cases per 100,000 population annually compared to 150–400 incident cases per 100,000 in high TB burden countries (WHO, 2019). Malnutrition, crowded living and work conditions and a lack of access to diagnosis and treatment contribute to a continued high disease burden in poor countries (Lonnroth et al., 2009). TB incidence is declining slowly at 1.6% per year globally but isoniazid and now rifampicin-resistant TB is increasingly reported (GBD Tuberculosis Collaboratoras, 2018) and the WHO’s “End TB Strategy” target of >4% sustained annual decline in incidence of new cases by 2,030 seems unlikely to be met (Furin et al., 2019).

Effective antimicrobial treatment for TB typically includes at least an intensive initial 2 months of therapy with four first-line drugs (isoniazid, rifampicin, pyrazinamide, and ethambutol) followed by continuation phase with isoniazid and rifampicin for 4 months (Nahid et al., 2016; WHO, 2017b), the efficacy of which is usually monitored with repeated sputum smears, cultures and chest X-rays. Drug toxicity is not uncommon and the duration of therapy makes compliance difficult (Pai et al., 2016). MDR TB is steadily increasing in endemic countries (Dheda et al., 2017) and the emergence of extremely drug resistant TB (XDR-TB), for which there is almost no effective treatment, creates a pool of patients actively transmitting untreatable strains (Pietersen et al., 2014). The Bacillus Calmette-Guerin (BCG) vaccine is used worldwide mainly to prevent life-threatening tuberculosis in infants and young adults but is not an effective eradication strategy (Abubakar et al., 2013). There are other candidate vaccines in development with better efficacy profile in newborns and children, as well as adolescents and adults (Pai et al., 2016), but none are widely available.

There is considerable interest in mycobacteriophages for treatment and control of TB. They have been tested against MDR and XDR strains (Hatfull, 2014) but early animal studies met with mixed results (Mankiewicz and Beland, 1964; Sula et al., 1981) and there are no human trials as yet. some benefits were observed in guinea pigs with disseminated TB (Zemskova and Dorozhkova, 1991) but there are concerns regarding phage penetration to attack intracellular bacilli or those deep within granulomatous lesions (Hatfull, 2014). Use of non-virulent bacteria to deliver phage payloads into macrophages led to a significant reduction in viable intracellular bacilli in experimental animals (Broxmeyer et al., 2002) but the clinical applicability of this approach needs further evaluation.

Prospects for phage prophylaxis of TB contacts may be better. Inhaled bacilli from exposure should be easily accessible to phages introduced directly into the lungs in high concentrations, perhaps reducing risk of resistance evolution among small populations of targeted bacteria (Vehring, 2016) and this approach appeared to reduce the pulmonary MTB burden in mice up to 3 weeks after challenge (Carrigy et al., 2019). Aerosolised phage delivery into the lungs may be the optimal route (reviewed by Abedon, 2015) but particle diameter and tolerance of physical, osmotic and thermal stress are important considerations (Hoe et al., 2013).

The natural course of TB is indolent and subclinical with most disease transmission in high-burden countries unrecognized until recrudescent (secondary) disease develops, often decades later. Phage therapy may have value as a prophylactic regimen in recent exposure populations but studies would probably need to be randomized as adjunctive therapy (with standard agents) and surrogate (e.g., serological) markers may be needed to avoid decades of follow-up. Phage prophylaxis and therapy may be most valuable in severe and/or XDR-TB where need is urgent and response relatively easily measured.

## Discussion

Phage therapy is increasingly re-emerging as a viable therapeutic option against serious bacterial infections. Notwithstanding the long experience in parts of Europe and the numerous anecdotes of successful phage therapy for human infection (d’Herelle, 1931; Summers, 2001; Abedon et al., 2011; McCallin and Brüssow, 2017; Gordillo Altamirano and Barr, 2019), it remains poorly accepted in Western medicine.

Recently, there has been a noticeable increase in compassionate use of phage therapy to treat serious bacterial infections and the results are promising (Maddocks et al., 2019; Aslam et al., 2020; Cano et al., 2020; Petrovic Fabijan et al., 2020b). However, robust clinical trials are very few and mostly unsuccessful (Sarker et al., 2016; Jault et al., 2019; Leitner et al., 2020).

There are some important challenges to the progress of phage therapy through the existing regulatory frameworks, most prominent being the scarcity of essential data from human therapy. Phage(s) selection, optimal route of administration and dosage, the relative benefit of single vs. multiple phages and/or combinations with antibiotics all remain as questions that have yet to be decided. Pharmacokinetic and pharmacodynamic properties are generally regarded as unpredictable and must be better defined (Nilsson, 2019). The *in vivo* co-evolution of phages and their target bacteria and the potential interference of human immune system further complicate phage therapy, and the fact that compassionate use cases typically utilize phage/s as adjuvants to antibiotic/s makes it more difficult to attribute efficacy. The emergence of phage-resistant bacteria may be countered by using multiple effective phages in a cocktail (Pirnay and Kutter, 2020) and this popular approach means that complex multi-phage dynamics must also to be considered.

Oral administration is an appealing option, especially for enteric diseases, with the key advantage of greater simplicity of manufacture. Safety of phage therapy has been well demonstrated for suitable preparations but oral bioavailability and dosing kinetics are not well understood (Bruttin and Brussow, 2005; Sarker et al., 2017; Petrovic Fabijan et al., 2020b). The limited data that are available suggest that bioavailability may be adequate for oral dosing in animals (Watanabe et al., 2007; Miedzybrodzki et al., 2017) and humans (Weber-Dabrowska et al., 1987).

Aerosol delivery of phages into lungs is also relatively simple (Abedon, 2015; Maddocks et al., 2019) but GMP-grade preparation for nebulization (as for IV administration) remains a financial and logistical challenge.

Much of the current demand for phages to treat major developing country diseases relies on goodwill to meet it. Organizations such as “Phages for Global Health” raise awareness and educate/train laboratory and healthcare staff and build capacity in developing countries while biotech ventures such as “PhagePro” work to develop phage-based products to treat cholera.

The willingness of phage laboratories in developed countries to donate therapeutic candidate phages are a boon for physicians in developing countries and organizations such as Phage Directory^1^ work to facilitate access to these but a sustainable approach must be developed.

Coordinated efforts are needed from international health organizations to properly evaluate the potential role of phage therapy and identify potential candidates for properly designed trials.

## Author Contributions

AK, RCYL, and JRI conceived and drafted the manuscript. All authors read and approved the final manuscript.

## Conflict of Interest

The authors declare that the research was conducted in the absence of any commercial or financial relationships that could be construed as a potential conflict of interest.

## Footnotes

1 https://phage.directory/
